# Apical Trafficking Pathways of Influenza A Virus HA and NA via Rab17- and Rab23-Positive Compartments

**DOI:** 10.3389/fmicb.2019.01857

**Published:** 2019-08-13

**Authors:** Ryota Sato, Takashi Okura, Madoka Kawahara, Naoki Takizawa, Fumitaka Momose, Yuko Morikawa

**Affiliations:** ^1^Graduate School for Infection Control, Kitasato Institute for Life Sciences, Kitasato University, Tokyo, Japan; ^2^Laboratory of Basic Biology, Institute of Microbial Chemistry, Tokyo, Japan

**Keywords:** influenza, HA, NA, apical trafficking, Rab family protein, post-TGN

## Abstract

The envelope proteins of influenza A virus, hemagglutinin (HA) and neuraminidase (NA), play critical roles in viral entry to host cells and release from the cells, respectively. After protein synthesis, they are transported from the *trans*-Golgi network (TGN) to the apical plasma membrane (PM) and assembled into virus particles. However, the post-TGN transport pathways of HA and NA have not been clarified. Temporal study by confocal microscopy revealed that HA and NA colocalized soon after their synthesis, and relocated together from the TGN to the upper side of the cell. Using the Rab family protein, we investigated the post-TGN transport pathways of HA and NA. HA partially colocalized with AcGFP-Rab15, Rab17, and Rab23, but rarely with AcGFP-Rab11. When analyzed in cells stably expressing AcGFP-Rab, HA/NA colocalized with Rab15 and Rab17, markers of apical sorting and recycling endosomes, and later colocalized with Rab23, which distributes to the apical PM and endocytic vesicles. Overexpression of the dominant-negative (DN) mutants of Rab15 and Rab17, but not Rab23, significantly delayed HA transport to the PM. However, Rab23DN impaired cell surface expression of HA. Live-cell imaging revealed that NA moved rapidly with Rab17 but not with Rab15. NA also moved with Rab23 in the cytoplasm, but this motion was confined at the upper side of the cell. A fraction of HA was localized to Rab17 and Rab23 double-positive vesicles in the cytoplasm. Coimmunoprecipitation indicated that HA was associated with Rab17 and Rab23 in lipid raft fractions. When cholesterol was depleted by methyl-β-cyclodextrin treatment, the motion of NA and Rab17 signals ceased. These results suggest that HA and NA are incorporated into lipid raft microdomains and are cotransported to the PM by Rab17-positive and followed by Rab23-positive vesicles.

## Introduction

The envelope proteins of influenza A virus, hemagglutinin (HA) and neuraminidase (NA), play critical roles in viral entry to host cells and release from the cells, respectively. They are prototypes for type I and II transmembrane proteins, respectively, and have long been used for studies on posttranslational modifications of protein and intracellular membrane trafficking. HA is composed of two subunits, HA1 and HA2: HA1 is an N-terminal soluble outer unit of HA with several antigenic domains and a receptor-binding pocket, whereas HA2 is a C-terminal transmembrane unit enriched in helical structures with a membrane fusion peptide. Influenza A virus binds to a sialic acid receptor via HA1 and is internalized to endosomes, where virus-cell membrane fusion involving HA2 occurs (Knossow et al., 1984; Gamblin and Skehel, 2010; Hamilton et al., 2012; Sriwilaijaroen and Suzuki, 2012). NA is composed of four domains: the N-terminal cytoplasmic tail domain, a transmembrane domain, a stalk domain, and the C-terminal head domain with sialidase activity. NA cleaves sialic acid receptors, thereby liberating progeny virus particles (Wang et al., 2008; Gamblin and Skehel, 2010; Air, 2012). It is well known that HA forms a homotrimer, whereas NA forms a homotetramer.

Influenza A virus, when infecting polarized cells (e.g., respiratory epithelial cells and Madin-Darby canine kidney [MDCK] cells), newly synthesized viral components are targeted to the apical plasma membrane (PM), where virus particles assemble and bud (Mora et al., 2002; Nayak et al., 2009). This apical trafficking and budding are responsible for transmission of influenza A virus. During the synthesis of HA and NA proteins, glycosylation takes place in the endoplasmic reticulum (ER) and Golgi apparatus, and the proteins are then transported from the *trans*-Golgi network (TGN) to the apical PM (Barman and Nayak, 2000; Zhang et al., 2000; Ohkura et al., 2014). They have traditionally been thought to traffic directly to the PM after the TGN, but their post-TGN pathways remain poorly defined. In certain cell types, HA has been suggested to follow a transcytotic pathway (Casanova et al., 1991; Bonilha et al., 1997). In contrast, it has been shown that viral genomic RNA-nucleoprotein complex (vRNP) replicates in the cell nucleus (Resa-Infante et al., 2011; Zheng and Tao, 2013) and the newly synthesized vRNP, following its export to the cytoplasm, is transported to the apical PM side through Rab11-positive compartments, likely by apical recycling endosomes (ARE; Bruce et al., 2010, 2012; Amorim et al., 2011; Eisfeld et al., 2011; Momose et al., 2011; Chou et al., 2013; Kawaguchi et al., 2015; Vale-Costa and Amorim, 2017).

Recent intensive studies have discovered the biosynthetic, endocytic, recycling, and transcytotic routes for apical and basolateral proteins in epithelial cells (Rodriguez-Boulan et al., 2005; Bhuin and Roy, 2014). Several studies have indicated that apical or basolateral sorting in the biosynthetic route starts at the common recycling endosomes (CRE) soon after exiting the TGN. The apical proteins are further transported to the apical PM via ARE. CRE also serves as a sorting platform for proteins that are internalized into apical and basolateral sorting endosomes (ASE and BSE), also called apical and basolateral early endosomes (AEE and BEE) (Rodriguez-Boulan et al., 2005; Folsch et al., 2009). Several sorting signals and determinants have been identified (Rodriguez-Boulan et al., 2005; Weisz and Rodriguez-Boulan, 2009). The cargo protein sorting to the PM is often modified with N- or O-glycans and fatty acids, which may serve as apical sorting signals. It is well documented that glycosylphosphatidylinositol-linkages confer apical PM targeting to cargo proteins (Muniz and Riezman, 2016). The association of proteins with lipid rafts has also been suggested to direct apical sorting of cargo proteins (Simons and Ikonen, 1997). Lipid rafts are thick membrane microdomains enriched in bulky sphingolipids and cholesterol, and serve as sorting platforms for cargo proteins (Simons and Ikonen, 1997). Influenza HA and NA are N-glycosylated, and are well-known lipid raft-associated proteins which are transported to the apical PM (Barman and Nayak, 2000; Zhang et al., 2000; Ohkura et al., 2014). Although HA and NA are very often employed for the study of apical vesicular trafficking, which post-TGN pathways they follow and what transport vesicles they use have still not been elucidated.

Rab proteins are low molecular-weight GTPases, and are responsible for intracellular vesicle transport. Approximately 70 Rab proteins have been identified in humans: each is localized to a different intracellular membrane compartment and controls a unique transport pathway (Stenmark, 2009; Hutagalung and Novick, 2011; Bhuin and Roy, 2014). Since they distribute on transport vesicles and in their target compartments in the steady state, they have been used as markers for intracellular localization of cargo proteins. In epithelial cells, Rab17 and Rab25 are localized to the ARE and mediate vesicular trafficking to the apical and basolateral PMs (transcytotic route) (Hunziker and Peters, 1998; Zacchi et al., 1998; Casanova et al., 1999; Hansen et al., 1999; Leyt et al., 2007; Beaumont et al., 2011; Haobam et al., 2014). Rab15 is also localized to the ARE, but predominantly to the ASE/AEE, and controls the sorting of cargo proteins into recycling or degradation pathways (Zuk and Elferink, 1999, 2000). Rab11 is localized to the RE, characterized morphologically in tubular compartments, and plays a role in the recycling of internalized proteins back to the apical PM (Ullrich et al., 1996; Grant and Donaldson, 2009). Several studies including ours have suggested that apical trafficking of influenza vRNP is controlled by Rab11 and traverses RE (Amorim et al., 2011; Momose et al., 2011; Vale-Costa and Amorim, 2017), but our study has also suggested that HA is spatially segregated from Rab11-positive compartments (Momose et al., 2011). HA and NA play critical roles in the pathogenesis of influenza A virus and are constituents responsible for its particle budding. Since they are also used as apical marker proteins, their post-TGN trafficking pathways need to be clarified. Here, we used Rab family proteins and investigated the post-TGN pathways involving HA and NA transport to the upper side of the cell. Our results indicated that HA and NA traversed multiple and possibly sequential trafficking pathways to reach the upper PM.

## Materials and Methods

### Virus and Plasmids

A derivative of the influenza A/Puerto Rico/8/34 (PR8) virus, in which the amino acid sequence of HA was modified (H141Y and E142Q) so as to be recognized by the mouse anti-HA monoclonal antibody 12-1G6 (mAb12-1G6), has been described previously (Ohkura et al., 2014). The PR8 derivative virus grows with the same kinetics as the original PR8 virus (Ohkura et al., 2014), and was used as a wild-type virus in this study. The HA and NA genes of the derivative virus were cloned into the eukaryotic expression plasmid pCAGGS. For live-cell imaging, the HA and NA genes were placed upstream of the EGFP and mStrawberry (mSB) genes, respectively, and were subcloned into pCAGGS plasmid (referred to as HA-EGFP and NA-mSB).

The cDNAs of Rab family proteins were cloned downstream of the AcGFP gene of pCANeoAcGFP, a derivative of pCAGGS (Momose et al., 2011). The dominant-negative (DN) mutants of Rab15, Rab17, and Rab23 with amino acid substitutions have been described previously (Zacchi et al., 1998; Reiner and Nathanson, 2008; Jian et al., 2016) and were expressed as N-terminal FLAG-tagged fusion proteins.

### Cell Culture, Viral Infection, and DNA Transfection

Madin-Darby canine kidney cells were maintained in Dulbecco’s modified Eagle’s medium (Sigma-Aldrich) supplemented with 10% fetal bovine serum at 37°C under 5% CO_2_ concentration. MDCK cells were seeded in 12-well plates and were cultured for 12 h before DNA transfection and virus infection. The cells were infected with influenza A virus at a multiplicity of infection (MOI) of 3 in 200 μl of Opti-MEM I (Invitrogen) supplemented with 0.3% bovine serum albumin (BSA). One h post-infection (hpi), the cells were washed and incubated with fresh culture medium at 37°C.

DNA transfection was carried out using Lipofectamine LTX (Invitrogen) according to the manufacturer’s instructions with some modifications. After addition of the DNA-liposome complex to the cells, the plate was centrifuged at 250 × *g* for 5 min in order to synchronize protein expression (spin transfection). At 3 h post-transfection (hpt), the cells were washed and incubated with fresh culture medium at 37°C.

### Establishment of Stable Cell Lines Expressing AcGFP-Rab Proteins

Madin-Darby canine kidney cells were transfected with AcGFP-Rab expression plasmid using Lipofectamine 2000 (Invitrogen). The cells were selected in the presence of 800 μg/ml of G418 sulfate for 1 week posttransfection. G418-resistant cell populations were seeded in 96-well plates at 0.8 cell/well and were subjected to single cell cloning in order to establish stable cell lines. Expression of AcGFP-Rab was confirmed by fluorescent microscopy.

### Immunofluorescence Microscopy

Madin-Darby canine kidney cells were fixed with 3.7% paraformaldehyde in phosphate buffered saline for 10 min at 25°C and were permeabilized with 0.5% Triton X-100 (TX-100) for 10 min at 25°C. After blocking, the cells were incubated with primary antibodies, mouse anti-HA mAb12-1G6, sheep anti-NA antibody (Ab) (AF4858, R&D Systems), and/or rabbit anti-NP antibody and subsequently with secondary Ab conjugated with Alexa Fluor 488, 568, or 647 (Molecular Probes). Cell nuclei were stained with DAPI. The cells were observed with a laser scanning confocal microscope (TCS-SP5II, Leica Microsystems). Confocal images were acquired at 0.5 μm intervals from the top to the bottom of the cell. Reconstitution of xz images was processed using ImageJ software.

To evaluate the colocalization of HA with each Rab, the Pearson correlation coefficient was calculated using ImageJ software. Triple colocalization was similarly evaluated by ImageJ software. For quantitation of HA localization in RabDN-expressing cells, cells were incubated with mouse anti-HA mAb12-1G6 (Ohkura et al., 2014) and rabbit anti-FLAG Ab (F7425, Sigma-Aldrich). 50 antigen-positive cells were observed and patterns of antigen distribution in individual cells were analyzed. For quantitation of cell surface expression of HA, cells were fixed with 3.7% paraformaldehyde without permeabilization and were incubated with anti-HA mAb12-1G6 (Ohkura et al., 2014). After post-fixation and membrane permeabilization, the cells were incubated with rabbit anti-HA Ab (Sino Biological). Confocal images were acquired at 0.5 μm intervals as before. In each acquired channel, the sum of fluorescence intensity values of a z-stack was calculated as a z-projection image using the “sum slices” command of ImageJ software. Single cells were selected as region of interest (ROI), and the mean fluorescence intensity (MFI) in each channel was measured. Approximately 50 cells were observed from multiple fields of the same sample, and the relative MFIs of cell surface HA to total HA were calculated.

### Live-Cell Imaging

Madin-Darby canine kidney cells were seeded in ϕ3.5 cm glass bottom dishes and were co-transfected with HA-EGFP and NA-mSB expression plasmids. MDCK cells stably expressing AcGFP-Rab were similarly cultured in ϕ3.5 cm glass bottom dishes and were transfected with the NA-mSB expression plasmid. Before live-cell imaging, the culture medium was replaced with Dulbecco’s modified Eagle’s medium without phenol red (Life Technologies) supplemented with 10% FBS and the cells were placed in a microscope stage top incubation chamber (Tokai HIT, Japan). Live-cell imaging was performed at 12 hpt using a confocal microscope (IX71, Olympus Optical, Japan) equipped with an oil immersion objective (Plan Apo N, 60×, 1.42NA, Olympus Optical) and a microlens-enhanced Nipkow-disk confocal scanner unit (CSUX1, Yokogawa Electric, Japan). Sequential images with excitation at 488 and 568 nm were acquired at 1-s intervals for 100 s (100 exposures each for GFP and RFP) by an electron multiplying CCD camera (Luca, Andor Technology, United Kingdom). Bleach and contrast corrections of acquired images were performed using ImageJ software, and tracking of punctate fluorescent signals using MTrackJ plugin created by Eric Meijering^1^.

### Membrane Solubilization and Coimmunoprecipitation Assay

Madin-Darby canine kidney cells were seeded in 12-well plates and were infected with influenza A virus. At 9 hpi, cells were suspended in 100 μl of TNE buffer (50 mM Tris pH 7.5, 1 mM EDTA, and 150 mM NaCl) containing 1 mM DTT and protease inhibitors (Complete Mini cocktail, Roche). After brief sonication, the cells were treated with 1% TX-100 at 4°C or 37°C for 30 min and were centrifuged at 17,400 × *g* for 30 min at 4°C to separate the soluble and insoluble fractions. The fractions were analyzed by western blotting with anti-GFP mAb (clone GSN149, Sigma-Aldrich) and anti-caveolin1 rabbit Ab (H-20, Santa Cruz).

For coimmunoprecipitation, the fractions were mixed with 10 μg of anti-HA mAb12-1G6 for 90 min at 4°C and were subsequently mixed with protein G-Sepharose beads (GE Healthcare) pretreated with 1% BSA at 4°C for 60 min. Following several washes with TNE buffer containing 0.1% TX-100, the beads were boiled in SDS-PAGE sample buffer. The samples were subjected to SDS-PAGE followed by western blotting with anti-HA mAb12-1G6 and anti-GFP mAb. Chemiluminescent signals were detected using an Image Quant LAS500 (GE Healthcare) and were quantified using ImageJ software.

### Cholesterol Depletion

Cholesterol depletion was performed as described previously (Ohkura et al., 2014). MDCK cells were cultured in the presence of 16 μM lovastatin (Merck) for 12 h and then transfected with an NA-mSB expression plasmid. At 3 hpt, the transfection medium was replaced with fresh growth medium containing lovastatin. One hour before harvest or observation, 10 mM of methyl-β-cyclodextrin (MβCD) was further added to the culture medium.

### Statistical Analysis

Intergroup comparisons were performed with two-tailed, unpaired *t-*test. All *P-*values were considered significant if less than 0.05.

## Results

### HA and NA Were Cotransported in the Cytoplasm

**FIGURE 1:**
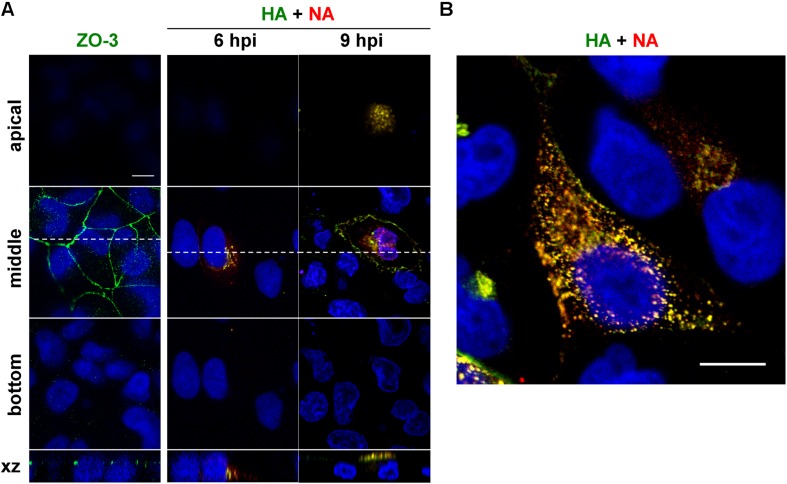
Relocation of HA and NA in polarized MDCK cells. Polarized MDCK cells were infected with an influenza A virus derivative containing H141Y and E142Q substitutions in HA (referred as to a wild-type virus) (Ohkura et al., 2014). **(A)** Polarization of MDCK cells was confirmed by staining with anti-ZO-3 mAb (green). At 6 and 9 hpi, cells were stained with anti-HA mAb (green), anti-NA Ab (red) and cell nuclei (blue) were stained with DAPI. Confocal images in the xy planes at the apical, middle, and bottom positions and reconstituted images in the xz planes were shown. Dashed lines in xy images indicate the positions of xz images. All images were taken at the same magnification. **(B)** Enlargement of the confocal xy images. Scale bar, 10 μm.

Time course study was performed to understand the overall kinetics of intracellular trafficking of HA and NA in cells infected with influenza A virus (Figure 1). Polarized MDCK cells were infected with a derivative of the influenza A/Puerto Rico/8/34 (PR8) virus and were immunostained with anti-HA mAb12-1G6 (Ohkura et al., 2014) and anti-NA Ab at 6 and 9 hpi. Polarization of MDCK cells was confirmed by staining with anti-ZO-3 mAb (Figure 1A). Serial confocal z sections of the cells were collected at 0.5 μm intervals. Confocal images in the xy plane (the top, middle, and bottom sections of the cell) indicated that at 6 hpi, HA and NA were observed at the perinuclear region of the middle sections but neither of them were seen in the apical or basolateral sections (Figure 1A). Soon after this time point, they colocalized and distributed in the cytoplasm (Figure 1B), and at 9 hpi, they accumulated at the apical section (Figure 1A). Few or no HA and NA antigens were distributed at the bottom sections throughout the period observed. These results suggested that HA and NA encounter each other relatively soon after protein synthesis, and were cotransported to the apical side. Such distribution kinetics was consistent with a previous study (Ohkura et al., 2014).

### Certain Rab Proteins Were Accompanied by HA Trafficking

**FIGURE 2:**
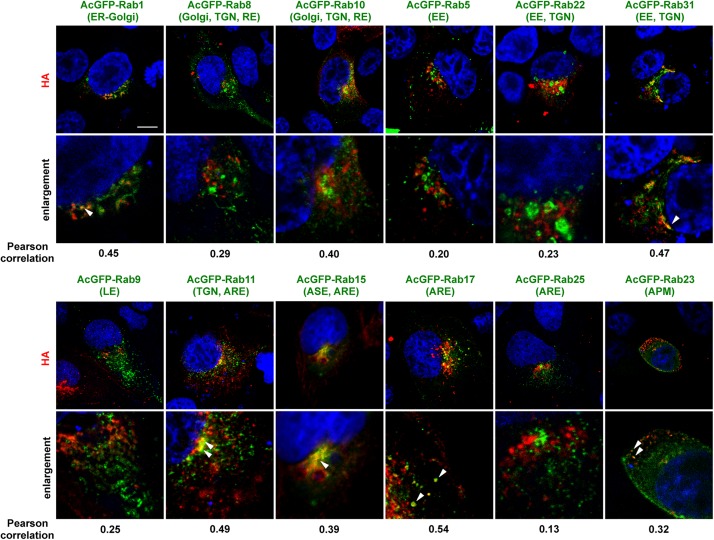
Colocalization of AcGFP-Rab proteins with HA. Polarized MDCK cells were transiently transfected with AcGFP-Rab1, Rab5, Rab8, Rab9, Rab10, Rab11, Rab15, Rab17, Rab22, Rab23, Rab25, or Rab31 expression plasmids and then infected with influenza A virus. At 6 hpi, cells were stained with anti-HA mAb (red) and cell nuclei (blue) were stained with DAPI. Confocal images in the xy planes were shown. ER, endoplasmic reticulum; TGN, *trans*-Golgi network; EE, early endosome; LE, late, endosome; ARE, apical recycling endosome; ASE, apical sorting endosome; APM, apical plasma membrane. Arrowheads indicate colocalization of HA and AcGFP-Rab. All images were taken at the same magnification. Scale bar, 10 μm. The Pearson correlation coefficient between HA and each Rab was also shown.

An initial study was conducted to identify the Rab proteins accompanying intracellular trafficking of HA. To this end, MDCK cells were transiently transfected with various AcGFP-Rab expression plasmids and then infected with the influenza A virus. At 6 hpi, the cells were subjected to immunostaining with anti-HA mAb and were observed by confocal microscopy (Figure 2). In polarized cells, the distributions of the Rabs are as follows: Rab1 is localized to ER-Golgi intermediates; Rab8 and Rab10 to Golgi and TGN in incompletely polarized cells but RE in fully polarized cells (Schuck et al., 2007); Rab5 to early endosome (EE); Rab22 and Rab31 to EE and TGN; Rab9 to late endosome (LE); Rab11 to TGN in incompletely polarized cells but ARE in fully polarized cells; Rab17 and Rab25 to ARE; Rab15 to ASE/AEE and ARE; and Rab23 to the apical PM (Stenmark, 2009; Bhuin and Roy, 2014). Colocalization efficiency of HA with Rab proteins were evaluated by Pearson correlation coefficient (PCC). HA partially colocalized with Rab1 and Rab31 in the biosynthetic pathway (PCC: 0.45 and 0.47), but not with Rab5 or Rab9 in the endocytic pathway (PCC: 0.20 and 0.25). In contrast, frequent colocalization of HA was observed with the Rab proteins involved in the recycling pathways. HA partially colocalized with Rab15 and Rab17 (PCC: 0.39 and 0.54), although not with Rab25 (PCC: 0.13). HA also partially colocalized with Rab10 (PCC: 0.40) but not with Rab8 (PCC: 0.29). Interestingly, colocalization of HA with Rab23 was also seen when the xy planes near at the apical position were observed. HA rarely colocalized with Rab11, except for the perinuclear region (PCC: 0.49). Thus, we selected Rab11, Rab15, Rab17, and Rab23 to investigate the post-TGN pathways of HA and NA in this study.

### HA and NA Colocalized With Rab15 and Rab17 in the Cytoplasm, and Later With Rab23 at the PM

**FIGURE 3:**
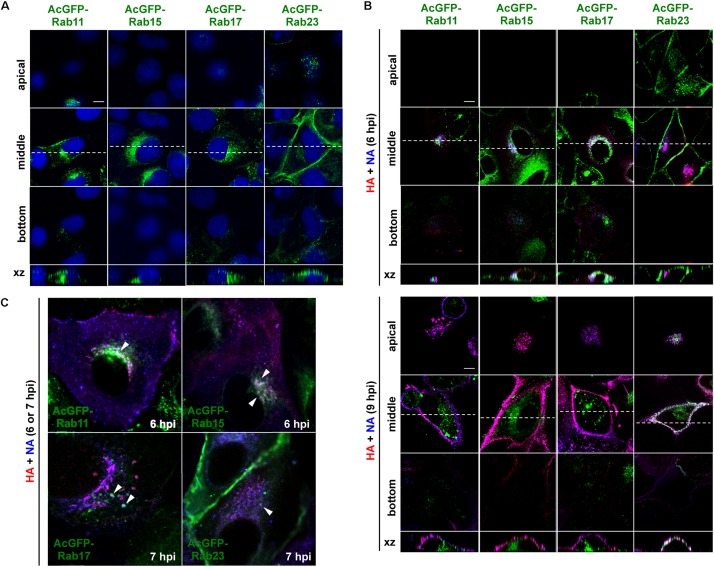
Localization of HA and NA in MDCK cells stably expressing AcGFP-Rab proteins. **(A)** MDCK cells stably expressing AcGFP-Rab proteins. MDCK cells were transfected with AcGFP-Rab11, Rab15, Rab17, and Rab23 expression plasmids and MDCK cells stably expressing AcGFP-Rab were established by single cell cloning. Cell nuclei were stained with DAPI (blue). **(B)** Localization of HA and NA. MDCK cells stably expressing AcGFP-Rab were infected with influenza A virus. At 6 and 9 hpi, the cells were stained with anti-HA mAb (red), anti-NA Ab (blue). Confocal images in the xy planes at the apical, middle, and bottom positions, and reconstituted images in the xz planes were shown. Dashed lines in xy images indicate the positions of xz images. All images were taken at the same magnification. Scale bar, 10 μm. **(C)** Enlargement of confocal xy images. Arrowheads indicate colocalization of HA, NA, and AcGFP-Rab.

Madin-Darby canine kidney cell lines stably expressing AcGFP-Rab15, Rab17, and Rab23 were generated by transfection of expression plasmids and subsequent single cell cloning (Figure 3A). For comparison, stable cells expressing AcGFP-Rab11 were similarly generated. Previous studies have indicated that epitope peptide and GFP tagging of Rab15, Rab17, and Rab23 do not perturb their intracellular localization (Zuk and Elferink, 1999, 2000; Boehlke et al., 2010; Haobam et al., 2014). Stably expressed AcGFP-Rab15 and Rab17 in MDCK cells were distributed in the perinuclear region, consistent with previous studies (Hunziker and Peters, 1998; Zacchi et al., 1998; Zuk and Elferink, 1999). A similar distribution pattern was observed for AcGFP-Rab11. In contrast, expression of AcGFP-Rab23 was predominantly observed at the PM. These stable cell lines were grown with a similar kinetics to the parental MDCK cells (data not shown) without aberrant cell morphology. To investigate the transport pathways involving HA and NA, the MDCK cells stably expressing AcGFP-Rab were infected with the influenza A virus. For a better cytoplasmic resolution, we used relatively flat cells grown on coverglasses, which may have been incompletely polarized. After immunostaining for HA and NA, their colocalization with each AcGFP-Rab was observed by confocal microscopy (Figure 3B). At 6 hpi, HA and NA were distributed among a fraction of Rab15-positive compartments in the middle sections of the cells. They were also partially localized with Rab17-positive compartments. In contrast, they were not distributed to Rab23-positive compartments at this time point (Figure 3B, upper panels). Only minor colocalization with Rab11 was observed (Figure 3C). At 9 hpi, large populations of HA and NA left from Rab15- or Rab17-positive compartments and were distributed to the PM, where they colocalized with AcGFP-Rab23 (Figure 3B, lower panels). The accumulation of HA and NA in the upper sections of the cells and their colocalization with Rab23 were more prominent at 12 hpi (data not shown). At 6 or 7 hpi, HA and NA often colocalized with Rab15 and Rab17, but less so with Rab23 in the cytoplasm (Figure 3C). Altogether, these results suggested that HA and NA were cotransported via the Rab15- and Rab17-mediated endocytic pathways, and subsequently through the Rab23-mediated pathway, and finally accumulated at the PM.

### NA Was Transported by Rab17-Positive and Rab23-Positive Vesicles

**FIGURE 4:**
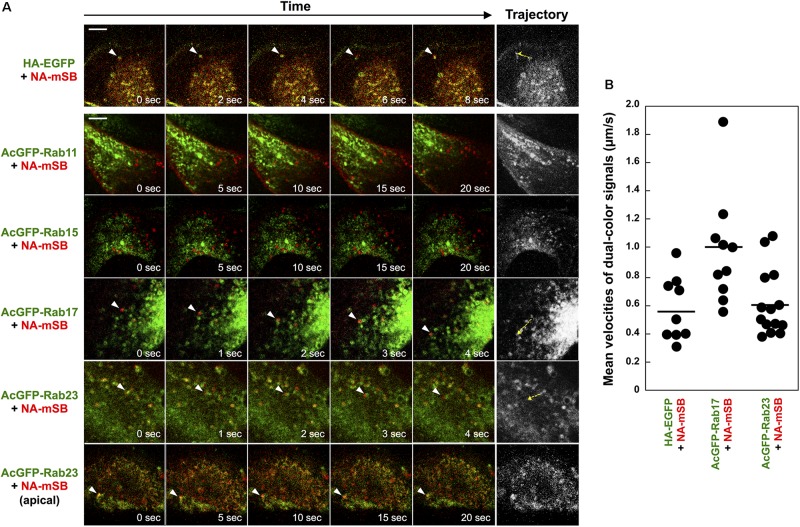
Cotrafficking of HA and NA and cotransport of NA by Rab17- and Rab23-positive vesicles. **(A)** Live-cell imaging of AcGFP-Rab proteins and NA-mSB. Parental MDCK cells were transiently cotransfected with HA-EGFP and NA-mSB expression plasmids. MDCK cells stably expressing AcGFP-Rab11, Rab15, Rab17, and Rab23 were transfected with an NA-mSB expression plasmid. Live-cell imaging was performed at 12 hpt using a confocal microscope. Dual color images were sequentially acquired at 1-s intervals and were processed by using ImageJ software. Sequential merged images were shown in Supplementary Movie S1. Time-split 5 images in each movie were shown. Arrowheads indicate cotrafficking signals. Examples of cotrafficking signals were shown as trajectories in most right panels (yellow dashed). Scale bar, 10 μm. **(B)** Velocities of NA-mSB cotransported with HA-EGFP, AcGFP-Rab17, or AcGFP-Rab23. Each dot indicates a vesicle analyzed. A horizontal line shows the mean velocity of analyzed vesicles in each group.

Although Rab15 and Rab17 are localized to RE and Rab23 to the PM and the endocytic pathway, they are not completely compartment-resident as they also mediate vesicular trafficking from such compartments (Zuk and Elferink, 2000; Evans et al., 2003; Boehlke et al., 2010; Beaumont et al., 2011; Hutagalung and Novick, 2011; Bhuin and Roy, 2014). Live-cell imaging was employed to explore whether HA and NA are transported on these Rab-positive vesicles (Figure 4). First, parental MDCK cells were cotransfected with HA-EGFP and NA-mSB expression plasmids, and were observed at 1-s intervals by confocal microscopy. Dual-color imaging revealed that the majority of fluorescent signals were double-positive and moved in the cytoplasm, confirming the cotrafficking of HA and NA (Figure 4A, first row images and Supplementary Movie S1, upper left). Tracking of the dual-color signals indicated that the mean velocity was 0.6 ± 0.2 μm/s, ranging from 0.3 to 1.0 μm/s. Since HA-EGFP and NA-mSB efficiently colocalized and moved together, NA-mSB was thereafter used for dual-color imaging with AcGFP-Rab. The addition of fluorescent protein to the NA C-terminus did not impair NA insertion into cell membranes or subsequent membrane transport (data not shown). MDCK cells stably expressing AcGFP-Rab were transfected with an NA-mSB expression plasmid. Confocal dual-color imaging revealed that NA-mSB signals overlapped with a fraction of AcGFP-Rab17 signals, and moved together in both the forward and backward directions (Figure 4A, fourth row images and Supplementary Movie S1, lower left). The mean velocity of the dual-color signals was 1.0 ± 0.4 μm/s, ranging from 0.6 to 1.9 μm/s, suggestive of microtubule-dependent vesicle transport. In contrast, mobile NA-mSB signals were not accompanied by AcGFP-Rab15 (Figure 4A, third row images and Supplementary Movie S1, upper right). Similarly, NA-mSB signals did not overlap with AcGFP-Rab11 signals (Figure 4A, second row images and Supplementary Movie S1, upper middle). When NA-mSB was observed in AcGFP-Rab23-expressing cells, the NA-mSB signals were found to colocalize with AcGFP-Rab23 in the cytoplasm and move together. The mean velocity of the dual-color signals was 0.6 ± 0.2 μm/s, ranging from 0.4 to 1.1 μm/s (Figure 4A, fifth row images and Supplementary Movie S1, lower middle). Interestingly, NA-mSB and AcGFP-Rab23 double-positive signals were static in the upper sections of the cells (Figure 4A, last row images and Supplementary Movie S1, lower right). These observations suggested that HA and NA were cotransported, at least via Rab17-positive and Rab23-positive vesicles to the PM.

### HA Partially Localized to Rab17 and Rab23 Double-Positive Vesicles

**FIGURE 5:**
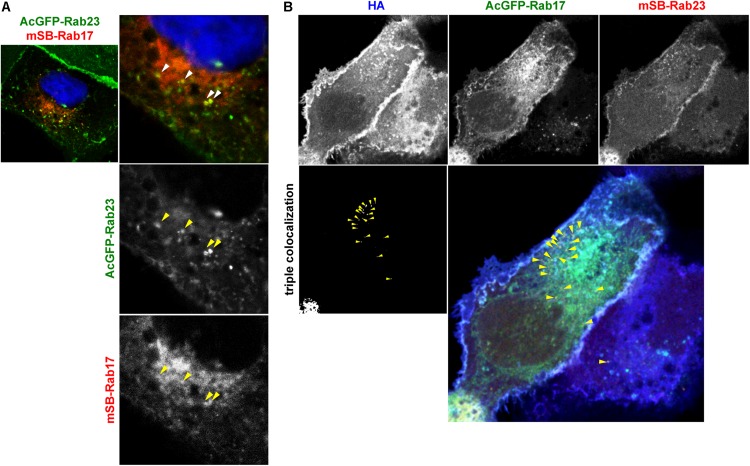
Localization of HA to Rab17 and Rab23 double-positive vesicles. **(A)** Coexpression of AcGFP-Rab23 and mSB-Rab17. MDCK cells stably expressing AcGFP-Rab23 were transfected with an mSB-Rab17 expression plasmid. Cell nuclei (blue) were stained with DAPI. Arrowheads indicate colocalization of AcGFP-Rab23 and mSB-Rab17. **(B)** Triple expression of AcGFP-Rab17, mSB-Rab23, and HA. Parental MDCK cells were cotransfected with expression plasmids for AcGFP-Rab17 and mSB-Rab23. After 12 hpt, cells were transfected with an HA expression plasmid and were stained with anti-HA mAb (blue). Each channel image was shown in gray and triple merged images were shown in gray and color. Arrowheads indicate colocalization of HA with AcGFP-Rab17 and mSB-Rab23 double-positive vesicles.

HA/NA colocalized with Rab17 at the perinuclear region and later colocalized with Rab23 at the PM (Figure 3). NA was transported via both Rab17-positive and Rab23-positive vesicles (Figure 4). To explore whether Rab17-positive and Rab23-positive compartments partially overlap, MDCK cells stably expressing AcGFP-Rab23 were transfected with an mSB-Rab17 expression plasmid (Figure 5A). Confocal microscopy revealed that a fraction of Rab23 colocalized with Rab17 in the cytoplasm. The results suggested that although the majority of Rab23 was accumulated at the upper PM, a fraction of Rab23 was distributed to the cytoplasm, overlapping with a Rab17-positive fraction. To further explore whether HA was localized to Rab17 and Rab23 double-positive vesicles, parental MDCK cells were cotransfected with AcGFP-Rab17 and mSB-Rab23 expression plasmids and were further transfected with an HA expression plasmid (Figure 5B). Triple color images were split into three channels and the combinations of two channel images were subjected to colocalization analysis. Results indicated that a fraction of HA colocalized with Rab17 and Rab23 double-positive vesicles, which were localized approximately midway between the perinuclear area and the cell periphery (Figure 5B).

### HA Transport Was Inhibited by Expression of Rab15DN and Rab17DN Mutants

**FIGURE 6:**
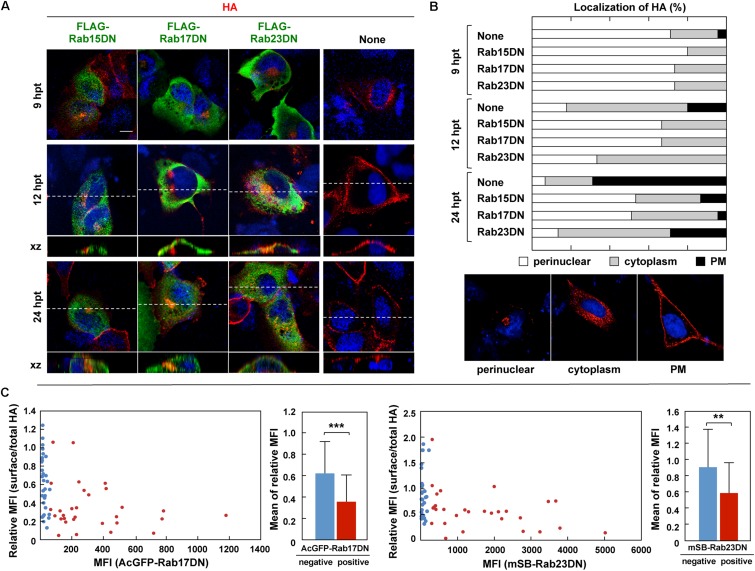
Aberrant localization of HA by coexpression with RabDN mutants. **(A)** Intracellular localization of HA in RabDN-expressing cells. Parental MDCK cells were transfected with expression plasmids for FLAG-tagged DN mutants of Rab15, Rab17, and Rab23. After 12 hpt, cells were transfected with an HA expression plasmid and were cultured for another 9, 12, and 24 h. At each time point, the cells were stained with anti-HA mAb (red) and anti-FLAG mAb (green). Confocal images in the xy planes were shown. Dashed lines in xy images indicate the positions of xz images. All images were taken at the same magnification. Scale bar, 10 μm. **(B)** For quantification of the HA localization, 50 antigen double-positive cells were subjected to HA distribution pattern analysis. Representative patterns of intracellular HA localization (perinuclear, cytoplasmic, and PM) were shown. **(C)** Cell surface expression of HA. Parental MDCK cells were transfected with expression plasmids for AcGFP-Rab17DN and mSB-Rab23DN mutants. After 12 hpt, cells were transfected with an HA expression plasmid and were cultured for 24 h. The cells were fixed without permeabilization and were incubated with mouse anti-HA mAb (for surface HA). After membrane permeabilization, the cells were stained with rabbit anti-HA Ab (for total HA). Confocal images were collected at 0.5 μm intervals in z-direction. In each acquired channel, the sum of fluorescence intensity values of a z-stack was calculated as a z-projection image by using ImageJ software. Single cells were selected as region of interest (ROI), and the MFI in each channel was measured. Approximately 50 cells were subjected to the calculation of the relative MFI of cell surface HA to total HA. In individual cells, the relative MFI of HA was plotted against the MFl of AcGFP-Rab17DN or mSB-Rab23DN. The cells expressing <5% of the maximum value of AcGFP/mSB MFI were considered as negative for Rab17DN/Rab23DN expression. The relative MFIs of HA were sorted into Rab17DN/Rab23DN-positive and negative groups and statistical analysis was performed with *t*-test. ^∗∗^*P* < 0.01; ^∗∗∗^*P* < 0.001.

The Rab GTPase cycles between a GTP-bound active form and GDP-bound inactive form (Hutagalung and Novick, 2011; Bhuin and Roy, 2014). Mutations that abolish the GTPase activity lead to constitutive activation, and mutations that block the exchange of GDT for GTP result in DN phenotypes. Both mutant types perturb the Rab functions. According to previous studies (Zacchi et al., 1998; Reiner and Nathanson, 2008; Jian et al., 2016), DN mutants of Rab15, Rab17, and Rab23 were constructed with a FLAG tag sequence and were coexpressed with HA in MDCK cells (Figure 6). Confocal microscopy showed that the RabDNs were diffusely distributed in the cytosol, consistent with previous studies (Zacchi et al., 1998; Reiner and Nathanson, 2008; Jian et al., 2016). When HA was expressed alone, three patterns of HA distribution (perinuclear; cytoplasm; PM) were usually observed, consistent with our previous study (Ohkura et al., 2014). HA was localized in the perinuclear area at 9 hpt, distributed in the cytoplasm at 12 hpt. and accumulated at the PM at 24 hpt (Figure 6B). In contrast, when HA was coexpressed with Rab17DN, the majority of HA was accumulated in the perinuclear area. Few HA antigens were seen at the PM (Figure 6A). Similar findings were observed for coexpression with Rab15DN. Interestingly, mild inhibition of HA transport was observed in Rab23DN-coexpressing cells. Punctate distribution of HA antigens in the cytoplasm was evident, but accumulation of HA at the PM was seen at low frequency (Figure 6A). For semi-quantification, we observed 50 HA and RabDN double-positive cells at three time points, and the numbers of cells with these distribution patterns were counted (Figure 6B). These results suggested that HA targeting to the PM was severely delayed when Rab15- and Rab17-mediated pathways were perturbed. These data also suggested that the PM transport of HA possibly involved at least two pathways: from the TGN to the cytoplasm and then to the PM.

To explore whether coexpression of Rab23DN indeed impaired PM targeting of HA, staining of cell surface HA was carried out (Figure 6C). mSB-Rab23DN and AcGFP-Rab17DN were coexpressed with HA in MDCK cells. Cell surface HA was immunostained with anti-HA mAb, and after membrane permeabilization, the cells were immunostained for the total cellular HA. When the ratio of the MFI of cell surface HA to that of total cellular HA in individual cells was plotted against the MFI of mSB-Rab23DN, an inverse correlation between the expression ratio of surface-to-total HA and the expression level of Rab23DN was observed (Figure 6C, right). A similar finding was observed for Rab17DN coexpression (Figure 6C, left). These results indicated that Rab23DN coexpression inhibited cell surface expression of HA, implying that Rab23-mediated pathway was responsible for HA expression on the cell surface. When the cells transfected with RabDNs were subjected to influenza virus infection, Rab17DN- and Rab23DN-expressing cells were relatively resistant to virus infection (Supplementary Figure S1), suggesting that these Rab proteins were also required for virus entry. A study has reported reduction of influenza virus replication by depletion of Rab17 (König et al., 2010).

### HA Interacted With Rab17 and Rab23 in Lipid Rafts

**FIGURE 7:**
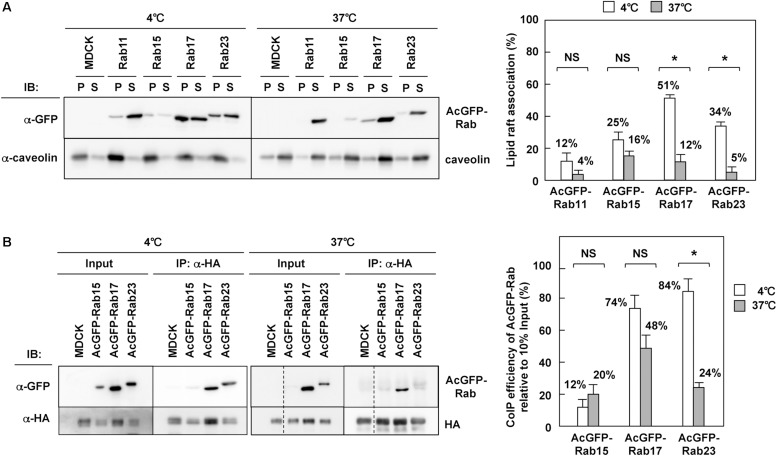
Interaction of HA with Rab17 and Rab23. **(A)** TX-100 solubilization of Rab. Parental MDCK and MDCK cells stably expressing AcGFP-Rab were lysed with TNE buffer containing 1% TX-100 at 4 or 37°C for 30 min, and were subjected to centrifugation at 30 min. P, insoluble pellet; S, soluble supernatant. The association of each AcGFP-Rab with lipid rafts was assessed by the ratio of the precipitate amount to the total amount (precipitate plus supernatant). **(B)** Coprecipitation of Rab with HA. Parental MDCK and MDCK cells stably expressing AcGFP-Rab were infected with influenza A virus. At 9 hpi, the cells were lysed with TNE buffer containing 1% TX-100 at 4 or 37°C for 30 min. After brief centrifugation, the cell lysates were incubated with anti-HA mAb and the precipitates were analyzed by western blotting using anti-GFP and anti-HA mAbs. The coimmunoprecipitation efficiency was calculated as the ratio of the coprecipitate to the 10% input. Representative data were shown with the means and standard deviations. ^*^P < 0.05; NS ≥ 0.05.

It is well known that influenza A virus HA and NA are associated with lipid raft microdomains (Barman and Nayak, 2000; Zhang et al., 2000; Scolari et al., 2016), which play pivotal roles in apical PM trafficking in polarized cells (Simons and Ikonen, 1997; Rodriguez-Boulan et al., 2005; Rog and Vattulainen, 2014). Previous studies on HA have shown that the disruption of HA association with lipid rafts by the use of non-raft mutants and treatment of cholesterol-lowering drugs cause a delay or block in TGN-to-apical PM trafficking of HA (Keller and Simons, 1998; Ohkura et al., 2014). Our results have indicated that HA and NA colocalize with certain fractions of Rab15, Rab17, and Rab23 (Figure 3), and are cotransported by at least Rab17-positive and Rab23-positive vesicles (Figure 4). The Rab protein contains prenyl (geranylgeranyl) tails, and a biochemical study with liposomes has suggested that prenylation excludes proteins from raft-like microdomains (Moffett et al., 2000). However, some studies suggested, although did not prove, the association of Rab17 and Rab23 with lipid rafts (Hansen et al., 1999; Emmer et al., 2010). Lipid rafts are characterized as non-ionic detergent-resistant membranes at 4°C (Brown and Rose, 1992; Edidin, 2003; Hancock, 2006). We solubilized MDCK cells stably expressing AcGFP-Rab with 1% TX-100 at 4°C, a condition under which lipid rafts were left intact, and separated detergent-insoluble membrane fractions by centrifugation. Western blotting with anti-GFP mAb showed that AcGFP-Rab17 and Rab23, similar to endogenous caveolin, were partially distributed to detergent-insoluble pellet fractions (Figure 7A). They were recovered to detergent-sensitive fractions when the cells were solubilized with 1% TX-100 at 37°C. The low recovery of AcGFP-Rab15 at 37°C may be attributed to protein degradation. The western blot bands were semiquantified using ImageJ software and the percent of precipitated AcGFP-Rab was compared between the 4°C and 37°C preparations. The results confirmed the association of AcGFP-Rab17 and Rab23 with lipid raft microdomains. In contrast, AcGFP-Rab11 was predominantly distributed to the detergent-soluble fractions at 4°C.

To explore whether HA interacted with Rab through lipid rafts, MDCK cells stably expressing AcGFP-Rab were infected with influenza A virus and were subjected to coimmunoprecipitation with anti-HA mAb. The coprecipitates were analyzed by western blotting with anti-GFP mAb and were semiquantified (Figure 7B). When membranes were solubilized with TX-100 at 4°C, AcGFP-Rab17 and Rab23 were efficiently coprecipitated with HA, suggesting that HA was associated with these Rab proteins through lipid raft microdomains. When membranes were solubilized at 37°C, the level of AcGFP-Rab23 coprecipitated with HA was significantly reduced, confirming the HA-Rab23 interaction through lipid rafts. AcGFP-Rab17, however, was efficiently coprecipitated with HA, even when membranes were solubilized at 37°C, suggesting that HA interacted directly with Rab17 in the lipid rafts. In contrast, little or no fraction of Rab15 was coprecipitated with HA, indicating that HA was not associated with Rab15 through lipid rafts at least in the steady state.

### Cholesterol Depletion Impaired Rab17- and Rab23-Mediated Trafficking of NA

**FIGURE 8:**
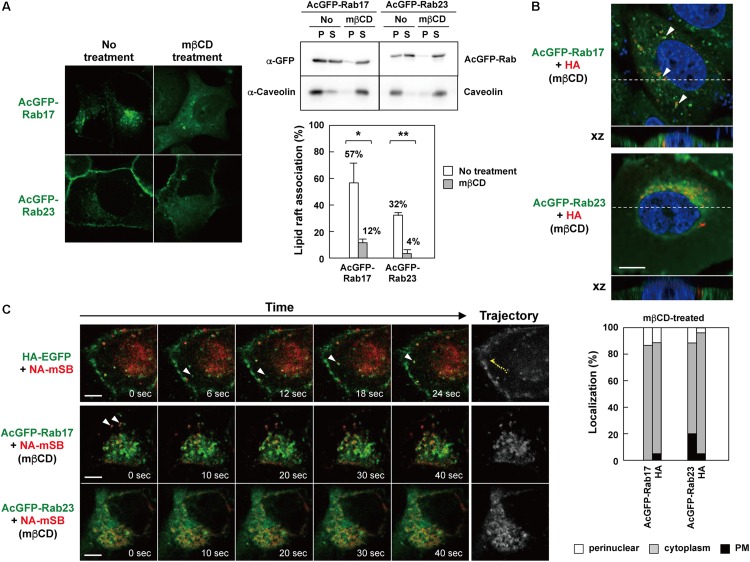
Impaired Rab17- and Rab23-mediated trafficking of NA by cholesterol depletion. **(A)** Cholesterol depletion. MDCK cells stably expressing AcGFP-Rab17 and AcGFP-Rab23 were treated with 16 μM lovastatin overnight and with additional 10 mM MβCD for 1 h. The cells were observed by confocal microscopy (left). The cells were lysed with TNE buffer containing 1% TX-100 at 4°C for 30 min, and were centrifuged at 4°C for 30 min (right). P, insoluble pellet; S, soluble supernatant. The association of AcGFP-Rab with lipid rafts was assessed by the ratio of the precipitate amount to the total amount (precipitate plus supernatant). Representative data were shown with the means and standard deviations. ^*^P < 0.05; ^∗∗^*P* < 0.01. **(B)** Confocal images of HA in cholesterol-depleted AcGFP-Rab17- and AcGFP-Rab23-expressing cells. Dashed lines in xy images indicate the positions of xz images. Both images were taken at the same magnification. Scale bar, 10 μm. Arrowheads indicate colocalization of HA and AcGFP-Rab17. For quantification of antigen localization, 20–25 cells were subjected to HA and AcGFP-Rab17/AcGFP-Rab23 distribution pattern analysis. **(C)** Live-cell imaging of NA-mSB in cholesterol-depleted AcGFP-Rab17- and AcGFP-Rab23-expressing cells. MDCK cells stably expressing AcGFP-Rab17 and AcGFP-Rab23 were pretreated with 16 μM lovastatin and then transfected with an HA **(B)** or NA-mSB **(C)** expression plasmid. The cells were treated with 16 μM lovastatin plus 10 mM MβCD for 1 h before paraformaldehyde fixation **(B)** or live-cell imaging **(C)**. Dual color live-cell images were sequentially acquired at 1-s intervals and were processed by using ImageJ software. Sequential merged images were shown in Supplementary Movie S2. Time-split 5 images in each movie were shown. For comparison, dual color live-cell images of HA-EGFP and NA-mSB in lovastatin/MβCD-untreated cells were shown. Arrowheads indicate colocalized signals. Cotrafficking signals were not seen in cholesterol-depleted cells. Scale bar, 10 μm.

It is well documented that the apical transport of influenza A virus HA and NA is inhibited or delayed by disruption of lipid rafts (Keller and Simons, 1998; Zhang et al., 2000; Ohkura et al., 2014). Our data have suggested that HA and NA are transported via Rab17-positive and Rab23-positive vesicles (Figure 4), and that they are associated with the Rab proteins through lipid rafts (Figure 7). To test whether the transport of HA/NA via Rab17-positive and Rab23-positive vesicles was impaired by the disruption of lipid rafts, cholesterol was depleted from cells coexpressing HA/NA and AcGFP-Rab17/AcGFP-Rab23. For cholesterol depletion, MDCK cells stably expressing AcGFP-Rab17 and Rab23 were treated with lovastatin and MβCD. Confocal microscopy revealed that in cholesterol-depleted cells, AcGFP-Rab17 was not distributed in the perinuclear region, but punctately scattered in the cytoplasm with diffuse cytosolic distribution. AcGFP-Rab23 was also diffusely distributed in the cytoplasm (Figure 8A, left). The majority of AcGFP-Rab17 and AcGFP-Rab23 in the cholesterol-depleted cells were recovered in the membrane-soluble fractions at 4°C (Figure 8A, right). Next, MDCK cells stably expressing AcGFP-Rab17 and AcGFP-Rab23 were pretreated with lovastatin and were transfected with an HA or NA-mSB expression plasmid. The cells were further treated with additional MβCD. At 12 hpt, HA was distributed in the cytoplasm, where some fractions exhibited colocalization with AcGFP-Rab17. For semi-quantification, we observed 20-25 HA and Rab double-positive cells, and the numbers of cells with three distribution patterns (perinuclear; cytoplasm; PM) were counted. Very little HA antigens were observed at the PM (Figure 8B). NA-mSB was similarly expressed in cholesterol-depleted AcGFP-Rab17/AcGFP-Rab23-expressing cells and was observed by live-cell imaging. Confocal dual-color imaging showed that NA-mSB still colocalized with AcGFP-Rab17, where the double-positive signals failed to move in the cytoplasm (Figure 8C and Supplementary Movie S2). Rather, they often formed relatively large aggregates. Similar findings were observed for cells stably expressing AcGFP-Rab23. The AcGFP-Rab23 vesicles carrying NA-mSB were static, although a considerably large fraction of AcGFP-23 was diffusely distributed in the cytoplasm. These data suggested that cholesterol plays a pivotal role in Rab17- and Rab23-mediated vesicular trafficking.

## Discussion

### Apical Trafficking Pathways Involving Influenza A Virus HA/NA and vRNP

Earlier studies with polarized MDCK cells have revealed that influenza A virus is assembled and buds at the apical PM, whereas vesicular stomatitis virus buds at the basolateral PM, (Rodriguez-Boulan and Sabatini, 1978; Rodriguez-Boulan and Pendergast, 1980). Since then, influenza A virus HA has been widely used as a tool for studying apical protein transport, and a study, using an adenovirus vector, has shown that HA is transported through ASE/AEE but not ARE in fully polarized MDCK cells (Cresawn et al., 2007). However, viral envelope proteins are often incorporated into non-cognate virus particles, i.e., pseudotyping, expression of HA alone needs to be tested. In polarized cells, several distinct subcellular compartments that apical proteins traverse en route to the apical PM have been identified (Rodriguez-Boulan et al., 2005; Folsch et al., 2009; Weisz and Rodriguez-Boulan, 2009). They include not only biosynthetic pathways but also endocytic and recycling pathways for apical proteins.

We investigated the trafficking pathways of influenza A virus HA using Rab family proteins. Rab11 mediates slow endocytic recycling through RE. Previous studies have shown that Rab11 is responsible for transport of influenza A virus vRNP (Amorim et al., 2011; Eisfeld et al., 2011; Momose et al., 2011). However, our present study indicated that HA and NA only colocalized with Rab11 at the perinuclear area (Figures 2, 3), consistent with the commonly believed notion that vRNP and HA/NA are transported via different pathways. We found that HA and NA partially localized to Rab17-positive compartments, most likely ARE. However, they were not localized to Rab25-positive compartments (Figure 2). Several studies have reported that both Rab17 and Rab25 are similarly involved in transcytosis of polymeric immunoglobulin receptors through lipid raft-containing ARE to the apical PM (Hansen et al., 1999; Leyt et al., 2007). The reason that HA preferentially follows a Rab17-mediated, rather than a Rab25-mediated pathway, is currently unknown, but our previous confocal studies have shown localization of a fraction of influenza vRNP to Rab25-positive compartments (Momose et al., 2011). It is possible that vRNP traverses Rab11- and Rab25-positive ARE whereas HA traverses Rab17-positive ARE, and that these viral components are not yet assembled into viral particles at the ARE during apical PM trafficking. In this study, we also found HA and NA partially localized to Rab15-positive compartments. However, live-cell imaging clearly indicated that NA was not transported via Rab15-positive vesicles, and that NA and Rab15 double-positive signals were static at the perinuclear area (Figure 4). It is possible that a fraction of NA (and HA) was transiently localized to Rab15-positive compartments and then was transported by another carrier vesicles. Alternatively, the NA fraction localized to Rab15-positive compartments may have been dead-end products for degradation, because Rab15, similarly to Rab5, predominantly localizes to the ASE/AEE and mediates sorting of cargos for recycling or degradation (Zuk and Elferink, 1999; Bhuin and Roy, 2014).

Our confocal microscopy revealed that HA and NA colocalized with Rab23 at the upper section of cells (Figures 2, 3), consistent with a previous study showing that Rab23 localizes to the PM and endocytic vesicles and controls vesicular trafficking between them (Evans et al., 2003). Rab23 has also been shown to be essential for formation of the primary cilium (Wang et al., 2006; Boehlke et al., 2010). Ciliogenesis involves microtubule organization and polarized membrane trafficking. Interestingly, screening of Rab proteins involved in ciliogenesis revealed that Rab17 and Rab23, in addition to Rab8-mediated microtubule formation, were required (Yoshimura et al., 2007).

### Sequential or Stepwise Trafficking Pathways of HA and NA

Our initial study revealed that HA and NA colocalized at an early time point of infection (6 hpi), when they were localized at the perinuclear region, and then relocated broadly to the cytoplasm (Figure 1). Live-cell imaging also revealed that the majority of HA signals were associated with NA signals and moved together in the cytoplasm. These data suggested that HA and NA were incorporated into the same membrane domains or vesicles at a relatively early stage of biosynthetic pathways, and were cotransported to the PM.

Time course observations with AcGFP-Rab-expressing MDCK cells indicated that HA and NA partially colocalized with Rab17 when they were present at the perinuclear region at 6 hpi. Later, they relocated to the upper side of the cell, where they colocalized with Rab23, but no longer with Rab17 (Figure 3). The experiments with RabDN have also suggested the stepwise pathways mediated by Rab17 and Rab23. When Rab17DN was overexpressed, HA was accumulated at the perinuclear region. In contrast, when Rab23DN was overexpressed, HA puncta were distributed in the cytoplasm, suggesting that Rab23DN did not completely block HA exit from the perinuclear compartments, but impaired subsequent PM targeting and/or cell surface expression (Figure 6). Together, these data suggest that apical transport of HA is composed of at least two chronological routes, one of which is mediated by Rab17, and the other by Rab23. It is tempting to speculate that HA/NA is targeted to the apical PM by transferring from Rab17-mediated pathways to Rab23-mediated pathways through vesicle fusion. In support of this hypothesis, we observed the colocalization of AcGFP-Rab23 and mSB-Rab17 in some frequency and that a fraction of HA was incorporated into Rab23 and Rab17 double-positive vesicles (Figure 5). Alternatively, HA/NA may have been sorted by maturation of membrane compartments. Recent studies have proposed a Rab cascade model in which one compartment transits to another by recruiting the GTPase activating protein for upstream Rab and the GTP exchange factor for downstream Rab (Hutagalung and Novick, 2011). Interestingly, we found that PM localization of AcGFP-Rab23 was severely impaired when Rab17DN, but not Rab23DN, was coexpressed. Perinuclear localization of AcGFP-Rab17 was not altered when either Rab17DN or Rab23DN was coexpressed (Supplementary Figure S2).

However, it is likely that HA can also use transport pathways other than Rab17-mediated pathways, since Rab17DN did not completely block, but rather delayed the PM targeting of HA (Figure 6B). Of note, our study found partial colocalization of HA with Rab10 (Figure 2), which is reported to localize to RE in fully polarized cells (Schuck et al., 2007). Similarly, a fraction of HA was observed at Rab11-positive compartments at an early time point (Figures 2, 3). Since Rab11 localizes to RE and mediates trafficking of many receptors and adhesion molecules (Kelly et al., 2012; Welz et al., 2014). It is possible that HA may be sorted into Rab10 and Rab11-positive vesicles and reach or meet the ARE containing Rab17.

Even though overexpression of Rab15DN similarly exhibited the transport blockage of HA, Rab15 was neither accompanied by NA-mSB movement nor associated with HA (Figures 4, 7). From these results, we presume that Rab15DN impaired the Rab17-mediated routes, not directly the transport of HA/NA, because both Rab15 and Rab17 were localized to ARE.

### The Dynamics of HA/NA Vesicles Containing Rab17 and Rab23 in a Cholesterol-Dependent Manner

Live-cell imaging displayed that in the cytoplasm, NA was transported by Rab17-positive vesicles (the mean velocity of 1.0 μm/s), and also by Rab23-positive vesicles (the mean velocity of 0.6 μm/s) (Supplementary Movie S1). These velocities are comparable with those demonstrated in microtubule-mediated transport of Influenza and Sendai vRNPs (Chambers and Takimoto, 2010; Amorim et al., 2011; Momose et al., 2011), vesicular stomatitis virus G protein (Hirschberg et al., 1998), and KIF1A (Lee et al., 2003). In contrast, NA-containing Rab23-positive vesicles were static at the upper side of the cell. This may imply that they reached their destination at the upper PM.

The association of Rab proteins with lipid rafts remains controversial, but our study indicated that relatively large fractions of Rab17 and Rab23 were distributed to detergent-insoluble membrane fractions (Figure 7). Since the lipid bilayer of cellular compartments are not uniform and are composed of many discrete microdomains, some fractions of Rab17 and Rab23 may be incorporated into lipid raft microdomains at the boundary between raft and non-raft microdomains if their interacting molecules are preferentially incorporated into raft microdomains. This is feasible especially for Rab17, because HA is likely to interact directly with Rab17 (Figure 7B). A previous study has shown transcytosis of IgA via Rab17-positive and lipid raft-enriched compartments (Hansen et al., 1999).

Cholesterol depletion and subsequent live-cell imaging indicated that NA became static upon disruption of lipid rafts, although NA still colocalized with Rab17 (Figure 8). Although it cannot be ruled out that cholesterol depletion affected general membrane dynamics, these data suggest that cholesterol was crucial for trafficking of HA/NA via Rab17- and Rab23-positive vesicles. A possible explanation for this trafficking defect would be the budding blockage of transport vesicles. A recent model for vesicle budding suggests that clustering of lipid raft microdomains at the cellular membrane induces membrane budding and generates transport vesicles (Schuck and Simons, 2004). Accordingly to this model, if cholesterol was depleted in the cytoplasm, lipid rafts would not be clustered, resulting in failure of vesicle budding even if HA and Rab coexist in the same membrane. It is possible that the aberrant cytoplasmic distribution observed after cholesterol depletion may have corresponded to budding-arrested structures from the cellular compartments. Alternatively, cholesterol depletion may have impaired cholesterol levels at the TGN-RE boundaries and failed to recruit raft-associated SNARE proteins, as previously suggested (Reverter et al., 2014). Note that cholesterol blockers, such as statins, do not reduce influenza incidence in humans, suggesting that suppression of immune responses rather than inhibition of influenza virus replication (Omer et al., 2016). Since cholesterol depletion impairs membrane dynamics such as vesicle budding, statins may block secretion of immunoglobulins and cytokines.

## Data Availability

The raw data supporting the conclusions of this manuscript will be made available by the authors, without undue reservation, to any qualified researcher.

## Author Contributions

YM conceived and supervised the study. RS, TO, and MK performed the experiments. RS and FM analyzed the data. RS, NT, FM, and YM discussed the data. RS and YM wrote the manuscript.

## Conflict of Interest Statement

The authors declare that the research was conducted in the absence of any commercial or financial relationships that could be construed as a potential conflict of interest.

## Footnotes

1 http://www.imagescience.org/meijering/software/mtrackj

## Supplementary Material

FIGURE S1 Failure of influenza A virus infection in Rab17DN- and Rab23DN-expressing cells. MDCK cells were transiently transfected with a FLAG-Rab15DN, FLAG-Rab17DN, FLAG-Rab23DN expression, or empty plasmid and then infected with influenza A virus. At 9 hpi, cells were stained with anti-FLAG mAb (green) and anti-NP Ab (red) and cell nuclei (blue) were stained with DAPI. The green and red channel images were also shown in gray. Confocal images in the xy planes were shown. Scale bar, 10 μm.

FIGURE S2 Failure of Rab23 localization to PM by coexpression with Rab17DN. Parental MDCK cells were transfected with FLAG-Rab17DN and FLAG-Rab23DN expression plasmids. After 12 hpt, cells were transfected with AcGFP-Rab17 and AcGFP-Rab23 expression plasmids and additionally FLAG-Rab17DN and FLAG-Rab23DN expression plasmids. The cells were stained with anti-FLAG mAb (red), and cell nuclei (blue) were stained with DAPI. The green and red channel images were also shown in gray. All images were taken at the same magnification. Scale bar, 10 μm.

MOVIE S1 Cotrafficking of NA-mSB and HA-EGFP, and NA-mSB transport by AcGFP-Rab17- and AcGFP-Rab23-positive vesicles. Parental MDCK cells were transiently cotransfected with HA-EGFP and NA-mSB expression plasmids. MDCK cells stably expressing AcGFP-Rab11, Rab15, Rab17, and Rab23 were transfected with an NA-mSB expression plasmid. For live-cell imaging, dual-color images with excitation at 488 and 568 nm were sequentially acquired at 1-s intervals for 100 s and were processed by using ImageJ software.

MOVIE S2 Impairment of AcGFP-Rab17- and AcGFP-Rab23-associated NA-mSB transport by cholesterol depletion. MDCK cells stably expressing AcGFP-Rab17 and AcGFP-Rab23 were pretreated with 16 μM lovastatin and then transfected with an NA-mSB expression plasmid. The cells were treated with 16 μM lovastatin plus 10 mM MβCD for 1 h before live-cell imaging. Dual color images were sequentially acquired at 1-s intervals for 100 s and were processed by using ImageJ software. For comparison, dual color live-cell images of HA-EGFP and NA-mSB in lovastatin/MβCD-untreated cells were shown.
