# Supplementary figures and images for: Apical Trafficking Pathways of Influenza A Virus HA and NA via Rab17- and Rab23-Positive Compartments
Source: Front Microbiol. 2019 Aug 13;10:1857. doi: 10.3389/fmicb.2019.01857 (PMC6700264; doi:10.3389/fmicb.2019.01857)

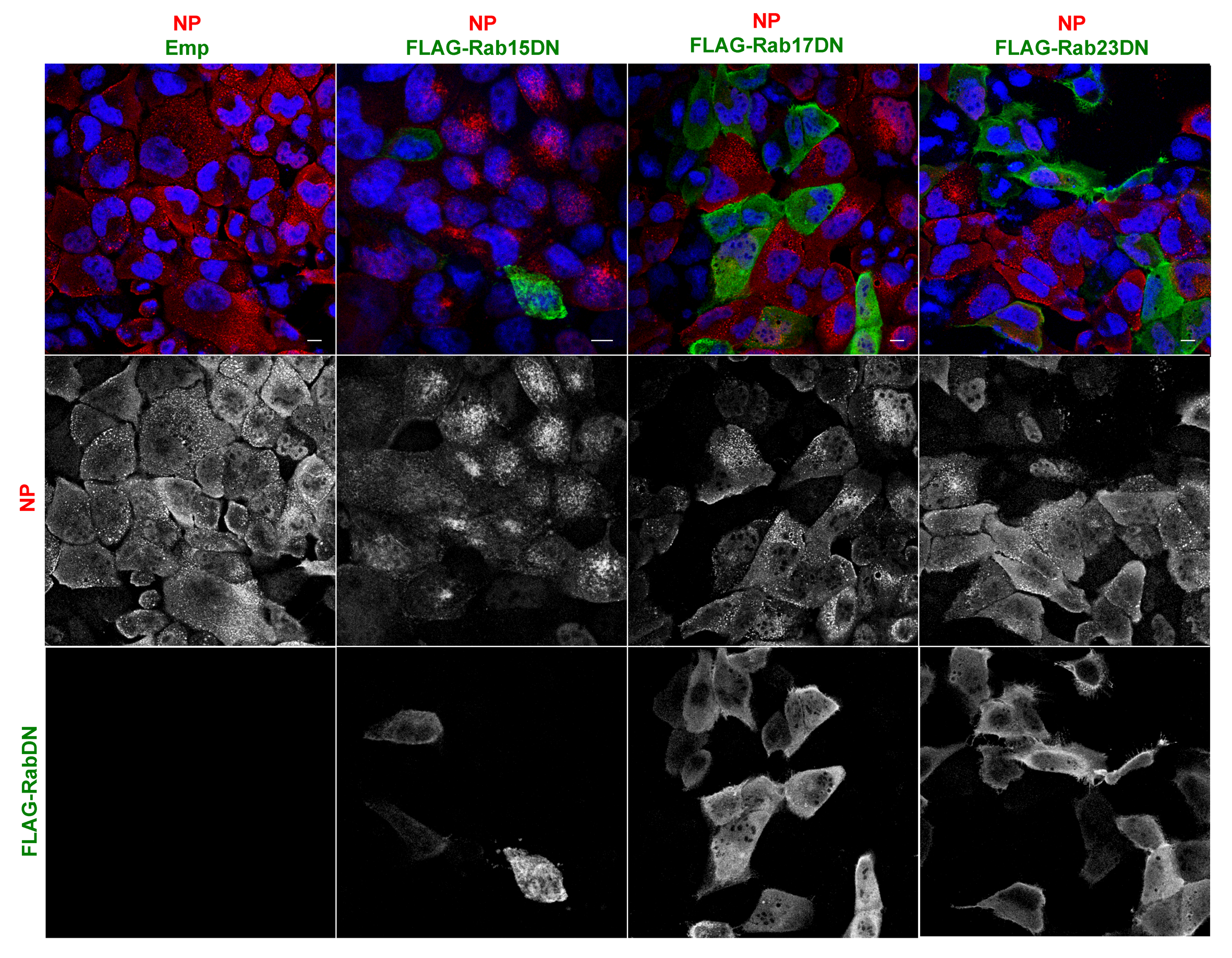

Supplement: FIGURE S1 — Failure of influenza A virus infection in Rab17DN- and Rab23DN-expressing cells. MDCK cells were transiently transfected with a FLAG-Rab15DN, FLAG-Rab17DN, FLAG-Rab23DN expression, or empty plasmid and then infected with influenza A virus. At 9 hpi, cells were stained with anti-FLAG mAb (green) and anti-NP Ab (red) and cell nuclei (blue) were stained with DAPI. The green and red channel images were also shown in gray. Confocal images in the xy planes were shown. Scale bar, 10 μm. [file image_1.TIF]

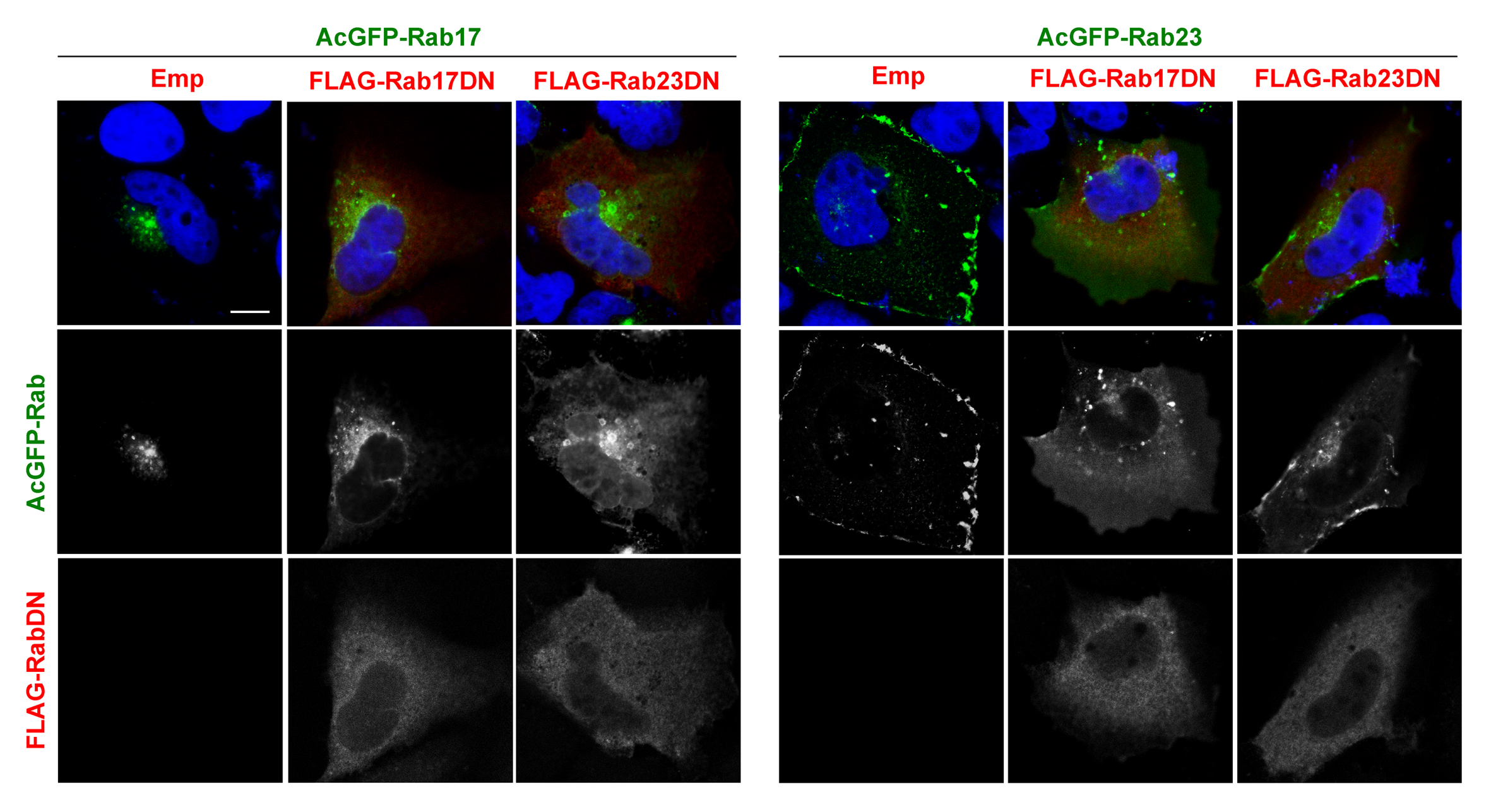

Supplement: FIGURE S2 — Failure of Rab23 localization to PM by coexpression with Rab17DN. Parental MDCK cells were transfected with FLAG-Rab17DN and FLAG-Rab23DN expression plasmids. After 12 hpt, cells were transfected with AcGFP-Rab17 and AcGFP-Rab23 expression plasmids and additionally FLAG-Rab17DN and FLAG-Rab23DN expression plasmids. The cells were stained with anti-FLAG mAb (red), and cell nuclei (blue) were stained with DAPI. The green and red channel images were also shown in gray. All images were taken at the same magnification. Scale bar, 10 μm. [file image_2.TIF]
